# Armed conflict as a determinant of children malnourishment: a cross-sectional study in The Sudan

**DOI:** 10.1186/s12889-020-08665-x

**Published:** 2020-04-19

**Authors:** Rihab Dahab, Laia Bécares, Mark Brown

**Affiliations:** 1 School of Social Sciences, Department of Social Statistics, University of Manchester, Humanities Bridgeford Street, Oxford Road, Manchester, M13 9PL UK; 2 University of Sussex, Brighton, UK

**Keywords:** Armed conflict, Malnutrition, Children under-5, The Sudan

## Abstract

**Background:**

Children’s nutritional status influences their physical, socioemotional and cognitive development throughout the life course. We aimed to determine the role of armed conflict on the prevalence of childhood malnourishment in The Sudan, and understand the underlying mechanisms using a framework based on the social determinants of health.

**Methods:**

We analysed cross-sectional data from the 2014-Sudan Multiple Indicator Cluster Survey (*n* = 14,081) to compare the prevalence of malnourishment in states undergoing armed conflict and states free of conflict. Four-level multilevel multivariate modelling was conducted to identify the contribution of the social determinants of malnourishment in explaining the role of armed conflict in child health, with conflict status as the central predictor and progressive adjustments for child-, household- and cluster- and state-level predictors.

**Results:**

Armed conflict is strongly associated with greater risk of severe and moderate underweight among children under-5. Adjusting for key social determinants of health reduced the strength of the association between armed conflict and risk of underweight, but there is statistical evidence of association between armed conflict and risk of severe underweight (OR: 1.60, 95%CI: 1.03–2.49 for the low intensity group).

**Conclusion:**

Conflict-exposed children are particularly vulnerable to malnourishment, and this association is mostly explained by key socio-demographic factors. With the prolonged political instability in The Sudan, sustainable nutritional interventions are necessary to ease hard conditions in conflict-exposed states, and also among disadvantaged families in conflict-free regions.

## Background

A striking decline in global rates of malnourishment among children under-5 has been observed recently, with stark inequalities experienced in the Global South. At the end of the Millennium Development Goals (MDGs) period (1990–2015), Sub-Saharan Africa (SSA) alone accounted for one third of all malnourishment among children worldwide [1]. Although demographic trends such as population growth of the region may be at play, this suggests that despite international progress, children in Africa are still at risk of serious health issues. The Sustainable Development Goals (SDGs), which replaced the MDGs in 2016, calls for *ending hunger, achieving food security and improving nutrition, and promoting sustainable agriculture* [2]. One noticeable difference between the two world’s frameworks for development is their nutritional-related targets. Whereas the MDGs placed all the emphasis on underweight prevalence only [1], target 2.2 (under Goal 2) of the SDGs indicates ending all forms of malnourishment by 2030 for all population, with a particular focus on children and the other vulnerable groups [2].

A country with particularly high rates of children malnourishment is The Sudan, where levels of malnourishment are among the highest in the world [3]. In 2014, underweight, stunted and wasted children were estimated at 33, 38.2 and 16.3%, respectively, compared to 14.6, 23.8 and 7% globally [4]. In addition, malnourishment among children under-5 has an increasing trend over the period between 2006 and 2014. For instance, underweight prevalence rose from 27% in 2006 to 29.7% in 2010. By 2014, this rate increased further to 33% [4]. This is due to factors including poverty, an inadequate healthcare system, high incidence of comorbidity and a fragile infrastructure [5]. While these features are common to many developing countries, an additional factor contributing to The Sudan’s alarming rates of malnourishment is the disrupting experience of armed conflict.

Malnourishment has short- and long-term adverse consequences in individuals [6]. The short-term damage can be observed as poor health and development, such as risk of morbidity, mortality and disability; whereas the long-term consequences present as poor psychological development and intellectual performance. Other long-term damages may be also seen in adulthood in the form of small body sizes, decreased economic productivity and increased vulnerability to serious health issues [6].

Although The Sudan’s long history of armed conflict has been associated with poor health among children [5], there is a dearth of research into its influence on their nutritional status. Apart from analysis often undertaken by international agencies to assess mortality rates from conflicts and to assist in estimating humanitarian relief needed by war-affected populations, to date there is not any academic research examining the association between conflict and malnourishment.

In addition, despite the association between armed conflict in Africa and poor nutritional status among children has been documented in some studies [7–9], the mechanisms behind this association have not been empirically examined, although several have been hypothesised. According to Agadjanian and Prata (2003), armed conflict has a direct negative impact on children’s health through the prevalence of malnourishment, and an indirect impact through attenuating healthcare services provided by governments, particularly the immunisation schedule [7]. Akresh et al. (2012) suggest two other possible mechanisms [8]. The first is the lack of security during conflict and its consequences on maintaining a durable food supply, one key determinant of malnourishment [10]. The second mechanism, which is common in Africa, is through the theft of households’ assets during conflicts. Loss of assets, particularly livestock, means an immediate threat to household food security and hence children’s food consumption [8], a direct determinant of malnourishment [10]. The indirect association between conflict and children’s nutritional outcomes is theorised to operate through socio-demographic factors [11]. During conflicts people are often forcibly displaced. With displacement, families are deprived of their basic needs including safe water, adequate sanitary and appropriate equipment to maintain hygiene. These provide breeding ground for infections [12], a main determinant of child morbidity and mortality [10]. Armed conflict also intensifies food insecurity. In conflicts, individuals cannot be committed to jobs due to lack of safety, which affects families’ affordability to buy food [11], one key determinant of malnourishment [10]. Shortage of healthcare services is also common during conflicts, either because medical facilities are not accessible due to insecurity or they become potential targets as a part of war strategy [13]. For children specifically, one of the most affected healthcare services during conflicts is the routine immunisation [7], an important determinant of malnourishment [10].

The aims of this study were twofold: we first aimed to assess whether armed conflict in The Sudan is associated with poor nutritional outcomes among children under the age of 5 years. We then examined the key socio-demographic factors (the social determinants of health) that are often exacerbated during conflict in order to understand the key mechanisms between armed conflict and childhood malnourishment.

## Method

### Study design and participants

We used data from The Sudan Multiple Indicator Cluster Survey (Sudan-MICS), a cross-sectional nationally representative household survey. At time of writing, the most up-to-date data available was that from 2014. The Sudan-MICS collects information about households and households’ members using stratified multistage cluster sampling design. The clusters were based on the enumeration areas used in the 2008 population census, classified into rural and urban areas of residence. The survey was mainly designed to produce precise estimates of certain indicators about the conditions of children and women at the national and sub-national level [14].

Data of children under-5 were collected from all mothers through face-to-face interviews. Of the 14,751 children under-5 who were listed in the completed household questionnaires, 14,081 (95.5%) mothers completed the survey [14], representing the sample size of the present study.

Permission to use data was obtained from the United Nations International Children’s Emergency Fund- Multiple Indicator Cluster Survey (UNICEF-MICS). The Sudan-MICS secures government permission and informed consent from all participants.

### Dataset

The outcomes in this study measure the severely or moderately underweight children under-5. Two dichotomous variables were created using syntax that was secured from the UNICEF-MICS team in New York. One variable assessed normal and severe underweight, and the other assessed normal and moderate underweight. Consistent with the World Health Organization (WHO) practices [15], severe underweight was defined as weight-for-age Z-score < − 3, moderate underweight was defined as − 3 ≤ Weight-for-age Z-score < − 2 and normal weight was defined as − 2 ≤ Weight-for-age Z-score < 2. We considered each measure separately in order not to mask the inequalities between well-nourished and severely malnourished children, and not to overestimate the differences between well-nourished and moderately malnourished children.

The predictors in this study are derived from the UNICEF model that conceptualises the determinants of malnourishment into immediate, underlying and basic [10] (see Additional File 1 for the construction of these variables). The central predictor measured intensity level/year of eruption of a conflict. We created this variable using the scale of the Heidelberg Institute for International Conflict Research (HIIK), which classifies conflicts intensity into three categories: low, medium, and high [16]. Although The Sudan was affected by seven different conflicts until 2014, we focused only on armed conflicts in Darfur (erupted in 2003); the eastern states (Red Sea, Kasala, and Gedaref), which erupted in 2005 and Blue Nile and South Kordofan (erupted in 2011). The intensity level of the conflicts in Darfur and South Kordofan and Blue Nile was classified as high, whereas that in the eastern states was classified as low. We aggregated this information into one variable by recoding the eighteen states of The Sudan to a four-category variable [conflict-free, low intensity/2005 (LI/2005), high intensity/2011 (HI/2011) and high intensity/2003 (HI/2003)]. Each category included, respectively, the eight states that are free of conflict, the eastern states that have been in conflict since 2005, Blue Nile and South Kordofan states that have been in conflict since 2011 and the five Darfur states that have been in conflict since 2003.

We also investigated the association between four levels of socio-demographic predictors and severe or moderate underweight: child-, household-, cluster- and state-level factors.

Child-level predictors included child age in years (a continuous variable), gender (boys, girls) and whether or not the child had diarrhoea in the 2 weeks preceding the survey.

Household-level predictors included mother’s age at childbirth (15–19, 20–29, 30–39, 40–49 years), mother’s educations (none, primary, secondary and above), father’s educations (none, primary, secondary and above, father is not in the household), wealth index (five quintiles), number of children under-5 living in the same household (a continuous variable), food consumption profile (FCP) and risk of contamination from sources of drinking water and types of sanitation facilities (RCWS). The FCP represented the classification of the household food consumption based on their weighted food consumption score. The score was computed using the Vulnerability Analysis and Mapping (VAM) seven-day recall period method that is recommended by the World Food Programme (WFP) [17]. Once this score was calculated, each household FCP was classified based on the WFP criteria into three categories: poor, borderline and acceptable. RCWS was created by summarising the categories of both the source of drinking water and the type of sanitation facility into two overarching “improved” and “unimproved” categories, according to the WHO/UNICEF guidelines [12]. Based on the assumption that improved sanitations are designed to separate water and sanitation facilities from external contamination, and to protect individuals from direct contact with contaminating pathogens [12], we combined the two sanitary services into three categories: no risk, medium risk and high risk. The “no risk” category measured whether both services were improved. The “medium risk” assessed if one of the services was improved (or unimproved). The “high risk” measured whether both services were unimproved.

The Sudan-MICS does not collect any data at cluster-level, with the exception of area of residence (urban-rural). We therefore created cluster-level versions of existing variables measuring children’s immunisation status, mothers’ education, fathers’ education, household wealth index and RCWS by aggregating them to respondent’s clusters. The three newly generated variables of maternal education, paternal education and wealth were subsequently developed into a principle component score (PCS) using principle component analysis. The revised variable, which represented an index for cluster-level socioeconomic status, accounted for the largest variation in the original data while being not correlated with other liner combinations [18]. Accordingly, our final analyses included four cluster-level variables: area of residence, immunisation coverage, cluster-level RCWS and cluster-level PCS. The Cronbach’s alpha for the cluster-level socioeconomic status index is 0.80.

We had one variable of state-level characteristics, which measured healthcare. We created this predictor with principal components analysis. We first generated two variables that assessed the healthcare provided per state (the proportion of hospital and doctors per 100,000 population) using information from the 2014 annual report of Ministry of Health [19]. We also used the 2013 economic review report of Ministry of Finance and Economic Planning [20] for creating a variable that measured the availability of national health insurance service per state. We finally calculated a state-level PCS, which transformed these variables into one component measuring the healthcare provided at state-level. The Cronbach’s alpha for the state-level healthcare index is 0.75. Summary statistics of the socio-demographic predictors are presented in Table A1 in Additional File 2.

The number of missing values of the predictors is very small, ranging from 0.01 to 2.9% of cases. But this increases for the outcome measures (19%). We conducted logistic regression analysis to examine the predictors of missing values. This analysis was undertaken on missing data for underweight prevalence taking into consideration all the predictors in this study. The results showed that the odd ratios of missingness in underweight children from the LI/2005 and HI/2003 states are higher compared to conflict-free states. When the other socio-demographic predictors were controlled for, the odd ratios of missing values for underweight prevalence in all classifications of conflict become smaller relative to conflict-free states. Accordingly, it is likely that the odds of underweight children from the LI/2005 and HI/2003 states are overestimated.

The small proportion of missing data for some of the predictors is presented in Tables A2, Additional File 3, and the association between missing values in outcome variable and all predictors is shown in Table A3, Additional File 3.

### Statistical analysis

We first performed bivariate analysis of the association between conflict status and underweight prevalence. To model the variance at community-, household-, and individual-level, and to consider intra-class correlations (ICCs), we conducted multilevel multivariate modelling (MLM) to analyse the two outcomes with random intercepts and fixed effects for each predictor. The use of MLM to investigate underweight among children is appropriate since observations within the same level are more likely to share similar characteristics, and failure to account for children hierarchical structure leads to underestimation of standard errors of regression coefficients and overstatement of statistical significance [21]. In this study, children from the same household are expected to have common characteristics either from their parents or the overall household. Also, children from a certain cluster or state may share common factors at community-level such as the availability of infrastructure and healthcare services. Accordingly, the unobserved variation in underweight prevalence could also be correlated to the different clustering level. MLM takes into consideration the effect of the variation between and within these different levels in the outcome [21].

The sample for the analyses of severe underweight included 8191 children (level 1), nested within 5821 households (level 2), nested in 673 clusters (level 3), nested in 18 states (level 4). The sample for the analyses of moderate underweight included 9080 children (level 1), nested within 6184 households (level 2), nested in 670 clusters (level 3), nested in 18 states (level 4). The modelling strategy was based on a step-wise adjustment: We first adjusted for armed conflict (Model 2), followed by adjustments for child-level predictors (Model 3). Model 4 further adjusted for household-level predictors and Model 5 additionally adjusted for cluster- and state-level predictors. Each model assumed that the association between conflict (and the other predictors) and each outcome varies by state, cluster and household. All analyses were performed using Stata 14 [22].

## Results

At national-level, rates of severe and moderate underweight were 12.6 and 21.4%, respectively (Table 1). Across the four groups of conflict status, the highest rates are observed in the low intensity states that have been in conflict since 2005 (the LI/2005 states) (16.8% for severe underweight and 22.9% for moderate underweight).

**Table 1:** Distribution of severe and moderate underweight and well-nourished children by conflict status

Prevalence of undernourishment	Conflict-free	Low intensity/ 2005	High intensity/ 2011	High intensity/ 2003	Overall
	*% (SE)*	*% (SE)*	*% (SE)*	*% (SE)*	*% (SE)*
**Underweight** ***(Weight for Age)***					
Severe	10.16 (0.442)	16.76 (0.941)	12.21 (0.759)	14.40 (0.616)	12.62 (0.312)
Moderate	19.88 (0.583)	22.86 (1.058)	22.27 (0.965)	22.34 (0.731)	21.39 (0.385)
Normal	69.96 (0.670)	60.38 (1.232)	65.52 (1.102)	63.26 (0.846)	65.99 (0.444)
**N**	**4683**	**1575**	**1859**	**3250**	**11,367**

Model 2 from Table 2 and Table 3 show the associations between armed conflict and severe and moderate underweight, respectively, before the adjustments for the socio-demographic measures. Except for the HI/2011 group, armed conflict is markedly associated with greater risk of severe and moderate underweight compared to the referent group (conflict-free states). Adjusting for child characteristics increases the risk of being underweight for all the groups of armed conflict (Model 3 from Table 2 and Table 3). The inclusion of household characteristics in Model 4 weakens noticeably the association between armed conflict and underweight. Accounting for all of the characteristics in Model 5 does not alter the association between armed conflict and moderate underweight (Table 3), whereas there is a statistical evidence of association between low intensity conflict and increased risk of severe underweight (OR: 1.60, 95%CI: 1.03–2.49) (Model 5, Table 2).

**Table 2:** Association of severe underweight with conflict status, child-, household- and cluster- and state-level characteristics

	Model (1) OR (95%CI)	Model (2) OR (95%CI)	Model (3) OR (95%CI)	Model (4) OR (95%CI)	Model (5) OR (95%CI)
**Conflict intensity level/year of eruption**					
Conflict-free (ref)					
Low intensity/2005 **(LI/2005)**		2.60^***^ (1.51–4.47)	2.73^***^ (1.52–4.92)	1.48^+^ (0.97–2.25)	1.60^*^ (1.03–2.49)
High intensity/2011 **(HI/2011)**		1.45 (0.80–2.64)	1.53 (0.80–2.93)	0.79 (0.51–1.25)	0.82 (0.50–1.32)
High intensity/2003 **(HI/2003)**		1.88^**^ (1.20–2.95)	1.97^**^ (1.21–3.21)	0.88 (0.61–1.27)	0.91 (0.58–1.44)
**Child age**			3.53^***^ (2.83–4.40)	3.51^***^ (2.82–4.37)	3.53^***^ (2.83–4.39)
**Presence of diarrhoea**					
No (ref)					
Yes			1.70^***^ (1.41–2.04)	1.65^***^ (1.37–1.98)	1.66^***^ (1.38–2.00)
**Mother education**					
None				3.03^***^ (2.15–4.26)	2.98^***^ (2.11–4.21)
Primary				2.06^***^ (1.52–2.80)	2.04^***^ (1.50–2.78)
Secondary + (ref)					
**Household wealth index quintile**					
Poorest				4.15^***^ (2.43–7.07)	3.10^***^ (1.69–5.69)
Second				4.24^***^ (2.58–6.96)	3.35^***^ (1.93–5.83)
Middle				3.13^***^ (1.99–4.94)	2.71^***^ (1.67–4.42)
Fourth				2.21^***^ (1.46–3.36)	2.07^***^ (1.35–3.16)
Richest (ref)					
**Food consumption profile (FCP)**					
Poor consumption				0.81 (0.50–1.29)	0.81 (0.51–1.29)
Borderline consumption				1.10 (0.86–1.41)	1.10 (0.86–1.41)
Acceptable consumption (ref)					
**Area of residence**					
Rural					1.65^**^ (1.22–2.24)
Urban (ref)					
**Intercept**	0.09^***^ (0.07–0.13)	0.06^***^ (0.05–0.09)	0.02^***^ (0.01–0.03)	0.003^***^ (0.001–0.006)	0.003^***^ (0.001–0.006)
**State-level variance**	0.24^*^ (0.10–0.57)	0.10 (0.03–0.30)	0.12 (0.04–0.35)	0.04 (0.01–0.19)	0.04 (0.01–0.20)
**Cluster-level variance**	0.86^***^ (0.63–1.18)	0.86^***^ (0.63–1.18)	1.00^***^ (0.73–1.36)	0.61^***^ (0.42–0.90)	0.57^***^ (0.38–0.84)
**Household-level variance**	1.86^***^ (1.28–2.70)	1.86^***^ (1.28–2.70)	2.13^***^ (1.48–3.06)	1.95^***^ (1.34–2.85)	1.92^***^ (1.31–2.81)
**State-level ICC**	0.04	0.02	0.02	0.01	0.01
**Cluster-level ICC**	0.18	0.16	0.17	0.11	0.10
**Household-level ICC**	0.47	0.46	0.50	0.44	0.43
**Observations**	8191	8191	8191	8191	8191
**LR chi2**		14.657	169.015	273.180	281.160

Values are relative odds ratios; 95% confidence intervals in brackets unless stated otherwise

The dependent variable: severe underweight vs. normal

^+^*p* < 0.10, ^*^p < 0.05, ^**^p < 0.01, ^***^p < 0.001

**Model (1):** the empty model

**Model (2):** adjusted for conflict intensity

**Model (3):** adjusted for conflict intensity + child-level predictors

**Model (4):** adjusted for conflict intensity + child-level predictors + household-level predictors

**Model (5):** adjusted for conflict intensity + child-level predictors + household-level predictors + cluster-level predictors + state-level predictors

**Table 3:** Association of moderate underweight with conflict status, child-, household- and cluster- and state-level characteristics

	Model (1) OR (95%CI)	Model (2) OR (95%CI)	Model (3) OR (95%CI)	Model (4) OR (95%CI)	Model (5) OR (95%CI)
**Conflict intensity level/year of eruption**					
Conflict-free (ref)					
Low intensity/2005 **(LI/2005)**		1.34^*^ (1.03–1.74)	1.33^+^ (1.00–1.78)	0.99 (0.78–1.25)	1.05 (0.84–1.32)
High intensity/2011 **(HI/2011)**		1.22 (0.94–1.59)	1.24 (0.92–1.68)	0.90 (0.71–1.14)	0.96 (0.76–1.20)
High intensity/2003 **(HI/2003)**		1.27^*^ (1.03–1.56)	1.29^*^ (1.02–1.63)	0.83^+^ (0.68–1.02)	0.96 (0.77–1.22)
**Child age**			2.24^***^ (1.93–2.59)	2.26^***^ (1.95–2.62)	2.26^***^ (1.95–2.62)
**Presence of diarrhoea**					
No (ref)					
Yes			1.42^***^ (1.25–1.62)	1.41^***^ (1.24–1.60)	1.41^***^ (1.24–1.60)
**Mother education**					
None				1.22^+^ (0.99–1.51)	1.17 (0.94–1.44)
Primary				1.12 (0.93–1.34)	1.10 (0.91–1.32)
Secondary + (ref)					
**Household wealth index quintile**					
Poorest				2.76^***^ (1.99–3.84)	1.97^***^ (1.35–2.87)
Second				2.43^***^ (1.80–3.29)	1.83^***^ (1.31–2.57)
Middle				2.05^***^ (1.56–2.69)	1.70^***^ (1.27–2.28)
Fourth				1.73^***^ (1.36–2.20)	1.58^***^ (1.24–2.01)
Richest (ref)					
**Food consumption profile (FCP)**					
Poor consumption				1.02 (0.74–1.41)	1.05 (0.76–1.45)
Borderline consumption				0.96 (0.81–1.15)	0.98 (0.82–1.16)
Acceptable consumption (ref)					
**Area of residence**					
Rural					1.56^***^ (1.29–1.88)
Urban (ref)					
**Intercept**	0.27^***^ (0.24–0.30)	0.23^***^ (0.20–0.27)	0.09^***^ (0.07–0.11)	0.05^***^ (0.03–0.06)	0.04^***^ (0.03–0.06)
**State-level variance**	0.03 (0.01–0.10)	0.01 (0.001–0.10)	0.02 (0.003–0.01)	0.003 (5e^−06^-1.53)	7.66e^−35^-
**Cluster-level variance**	0.31^***^ (0.23–0.43)	0.31^***^ (0.23–0.43)	0.36^***^ (0.27–0.50)	0.25^***^ (0.17–0.36)	0.22^***^ (0.14–0.33)
**Household-level variance**	0.48^***^ (0.26–0.86)	0.47^***^ (0.26–0.86)	0.59^***^ (0.35–1.01)	0.59^***^ (0.35–1.01)	0.57^***^ (0.33–0.98)
**State-level ICC**	0.01	0.003	0.004	0.001	1.88e-35
**Cluster-level ICC**	0.08	0.08	0.09	0.06	0.05
**Household-level ICC**	0.20	0.19	0.23	0.20	0.20
**Observations**	9080	9080	9080	9080	9080
**LR chi2**		7.702	216.864	303.887	329.704

Values are relative odds ratios; 95% confidence intervals in brackets unless stated otherwise

The dependent variable: moderate underweight vs. normal

^+^*p* < 0.10, ^*^p < 0.05, ^**^p < 0.01, ^***^p < 0.001

**Model (1):** the empty model

**Model (2):** adjusted for conflict intensity

**Model (3):** adjusted for conflict intensity + child-level predictors

**Model (4):** adjusted for conflict intensity + child-level predictors + household-level predictors

**Model (5):** adjusted for conflict intensity + child-level predictors + household-level predictors + cluster-level predictors + state-level predictors

When all predictors are adjusted for in Model 5, risk of severe and moderate underweight are associated with child’s age, presence of diarrhoea in the 2 weeks preceding the survey, poor household wealth index and living in rural areas. Mother’s education is only associated with severe underweight (Model 5, Table 2 and Table 3). The associations by individual predictors for severe and moderate underweight are shown in Table A4 and Table A5, respectively, in the additional file [see Additional File 4].

## Discussion

This study aimed to examine whether children affected by armed conflict in The Sudan are at risk of poor nutritional outcomes, and also to determine the mechanisms behind the association between armed conflict and child malnourishment. This is the first study to compare the role of armed conflict in The Sudan on the nutritional status of children. It is also the first study that uses appropriate modelling techniques to identify the determinants of malnourishment in a state of armed conflict. As mentioned earlier, discrepancies in children’s nutritional outcomes may be due to the various clustering level [21]. Therefore, by conducting MLM, this study not only compares the prevalence of malnourishment in states undergoing conflict against conflict-free states, but also identifies the association between the variations at the different levels and malnourishment. This is important as it provides accurate estimates and standard errors of malnourishment among children, and also reveals the mechanisms behind the association between conflict and children malnourishment [21].

Our findings show marked differences in the prevalence of malnourishment by conflict status. Armed conflict in the states classified as LI/2005 and HI/2003 are strongly associated with severe and moderate children malnourishment, with the greatest risk in the LI/2005 states. This association between conflict and underweight strengthens after adjustment for child characteristics. After accounting for all of the socio-demographic factors in the fully adjusted model, our findings show that the odds of the associations between conflict in high intensity states and severe and moderate underweight are reduced so that only children from the low intensity states are at risk of being severely underweight.

Our results are consistent with existing research from the few SSA studies that have reported a strong association between exposure to armed conflict and childhood malnourishment [7–9]. For instance, the two-year conflict between Eritrea and Ethiopia that broke out in 1998 led to a higher prevalence of malnourishment among children who were born during the war and were living in war-affected regions, compared to their counterparts in non-affected regions [8]. Another study conducted in Somalia has shown that both recent conflicts and longer-term conflicts are found to be associated with poor nutritional status among children [9]. That study has also found that longer-term conflicts have a stronger association with malnourishment relative to recent conflicts [9]. Although it is relevant to understand the role of conflict on children’s health, these studies failed to examine the impact of the differences between children themselves, which occur either because of variability between the households or the communities they live in, on the association between conflict and malnourishment. They therefore have missed the chance to obtain robust estimates and reliable results that contribute to our understanding of the association between armed conflict and childhood malnourishment.

Due to the lack of detailed information at the state-level, it is unclear why conflict in the low intensity states is associated with severe underweight when adjusting for all characteristics; however, according to the report of the 2015-Sudan Humanitarian Fund [23], these states received the lowest amounts of humanitarian aid as compared to other states in conflict. For instance, in 2015, the greatest amount of aid ($34.72 million) was received by the HI/2003 states (Darfur states), whereas the least ($1.36 million) was allocated to the LI/2005 states (Red Sea, Kasala and Gedaref), with the HI/2011 states (South Kordofan and Blue Nile) receiving $7.80 million [24]. It is possible that the uneven distribution of the humanitarian aid moderated the association between armed conflict and malnourishment in the high intensity states.

Our findings also show that when all predictors were controlled for, the odds of the associations between living in conflict states and severe or moderate underweight reduced compared to the odds of the associations between conflict solely and severe or moderate underweight. This provides support for current literature suggesting that there are indirect pathways in the association between conflict and malnourishment, which operate via children’s socio-demographic factors [7]. Among child-level predictors, our analyses show noticeable associations between the presence of diarrhoea before the survey and severe and moderate underweight. A cross-sectional analysis in Nigeria also found that recent incidence of diarrhoea was strongly associated with severe and moderate underweight among children [25]. Research has consistently documented that diarrhoea is the infectious disease most related to malnourishment [26–28]. For instance, Brown et al. (2013) described the relationship between diarrhoea and malnourishment as *complex* [29]. They explained that malnourished children experience diarrhoea frequently; also, severe episodes of diarrhoea contribute notably to causing growth retardation among children. Children who live in disadvantaged households or areas with low quality of services, such as unsafe drinking water and poor levels of hygiene and sanitation, are more vulnerable to enteric pathogens that cause greater risk of severe episodes of diarrhoea [30]. In addition to the poor hygiene and sanitation in conflict settings, families lose essential hygiene items during movement to more peaceful areas [31]. This is another factor that may increase risk of contamination and children’s vulnerability to infections, an immediate determinant of malnourishment [10]. Our results also report marked associations between child age and severe and moderate underweight, which has been found in other studies [32]. One factor that likely plays an essential role in this association is weaning period. Studies show that rates of malnourishment increase when children are introduced to solid food [33]. Children who were weaned at the recommended age of 4–6 months had lower risk of malnourishment than children who were weaned before or after the recommended age. The unsafe sources of weaning food and the unhygienic methods of food preparation increase children susceptibility to infectious disease [34], a direct determinant of malnourishment [10].

We also found that household wealth is a strong determinant of severe and moderate underweight. These results are consistent with previous research, which has also found that household economic status is strongly associated with severe and moderate underweight [35]. In conflicts, a sustainable family income may be threatened by insecurity [11]. Therefore, it is not surprising to find a clear association between household wealth and both indicators of underweight. Our findings do not support the assumption that household food security is a key determinant of malnourishment [10]. This is possibly because the variable measuring this factor (FCP) was generated using the VAM seven-day recall period method [17]. Vhurumuku suggests that this approach has limitations as it reflects household food consumption only over a period of single week [36]. It therefore does not show differences in food intake that are caused by crises [36]. Our results also showed a remarkable association between mother’s education and severe underweight, which is similar to the findings of Rahman [35]. The exact mechanism of the association between mother’s education and malnourishment during conflict is unclear in the literature, but one can expect maternal education may impact on children’s development during conflict through successfully navigating health services provided by humanitarian organisations, thus reducing infections due to lack of hygiene. Finally, we also found a strong association between living in rural areas and severe and moderate underweight among children. In The Democratic Republic of the Congo, malnourishment was also found to be remarkably higher in rural areas compared to urban [37]. Armed conflict adds an extra burden to basic services provided in rural settings [11]. The unsafe conditions during conflict, coupled with the poor quality of roads may also interrupt the flow of a sustainable food supplies to rural dwellers. Therefore, it is not unexpected to find a marked association between area of residence and malnourishment.

This study is not without limitations. First, we used cross-sectional data, which limited our ability to measure the casual effect of armed conflict (and the other determinants) on children’s underweight. Second, the number of missing values for the outcome measure (underweight) is large (19%). Our logistic regression analysis showed that it is likely that the odds of underweight children from the LI/2005 and HI/2003 states are overestimated. Finally, despite the richness of the data used in terms of variables that are suitable for investigating underweight, the measures used in this analysis did not fully explain the effect of household-level variations in levels of underweight among children. This is mainly related to the characteristics of the data used. For instance, the challenging condition of fieldwork during conflict is likely to negatively impact on data quality. Moreover, some measures used in analysing key determinants at household-level such as FCP and RCWS were recalculated from their original forms, so it is possible that the newly generated variables failed to capture the exact differences in malnourishment.

## Conclusion

This study is the first to document the association between armed conflict and poor nutritional outcomes among children under-5. Findings highlight the harm of armed conflict on children’s malnourishment and identify the mechanisms by which armed conflict operates. Conflict-exposed children are particularly vulnerable to malnourishment, and this association is mostly explained by socioeconomic factors. With the prolonged political instability in The Sudan, sustainable nutritional interventions are necessary to ease hard conditions in conflict-exposed states, and also among disadvantaged families in conflict-free regions.

Improving children’s nutrition is essential for achieving the Sustainable Development Goals, but consistent progress in different sectors needs to be made. In 1953, The Sudan was described as *a bright spot in a dark continent* [38]. Unfortunately, six decades of civil wars have contributed greatly in dimming this brightness. Policy makers must acknowledge that setting up advanced strategies to reduce the observed inequalities between states, and to advocate for education; particularly among girls is a priority. These indeed will be reflected in households’ economic conditions and ultimately in children’s health and wellbeing.

## Supplementary information

**Additional File 1:.** Appendix. Construction of the predictors.

**Additional File 2:.** Appendix. Summary statistics of the socio-demographic predictors.

**Additional File 3:.** Appendix. Missing data for some of the predictors and the association between missing values in outcome variables and all predictors.

**Additional File 4:.** Appendix. The associations by individual predictors for severe and moderate underweight.

## Data Availability

The data used in this study are available open access from online data repository available to registered users (https://mics.unicef.org/surveys).

## Abbreviations

MDGs: Millennium Development Goals
SSA: Sub-Saharan Africa
SDGs: Sustainable Development Goals
Sudan-MICS: The Sudan Multiple Indicator Cluster Survey
UNICEF-MICS: The United Nations International Children’s Emergency Fund- Multiple Indicator Cluster Survey
WHO: The World Health Organization
HIIK: The Heidelberg Institute for International Conflict Research
LI/2005: A low intensity armed conflict/has been ongoing since 2005
HI/2003: A High intensity armed conflict/has been ongoing since 2003
HI/2011: A High intensity armed conflict/has been ongoing since 2011
FCP: Food consumption profile
RCWS: Risk of contamination from sources of drinking water and types of sanitation facilities
VAM: The Vulnerability Analysis and Mapping
WFP: The World Food Programme
PCS: Principle component score
ICC: Intra-class correlation
MLM: Multilevel multivariate modelling
