# Supplementary material for: Armed conflict as a determinant of children malnourishment: a cross-sectional study in The Sudan
Source: BMC Public Health. 2020 Apr 19;20:532. doi: 10.1186/s12889-020-08665-x (PMC7168991; doi:10.1186/s12889-020-08665-x)
Supplement: Supplementary file 1 — Additional File 1:. Appendix. Construction of the predictors. [file 12889_2020_8665_MOESM1_ESM.docx]

**Additional File 1**

**Appendix**

The predictors in this study were derived from the UNICEF model, which conceptualises the determinants of malnourishment. They are divided, according to the pathway of their influence on nutritional status, into three different levels: immediate, underlying and basic. They also operate at various levels in a society, such as individual-, household- and community-level (1). In this study, these predictors were conceptualised as measuring nutritional status based on child, household, cluster and state characteristics.

As suggested by the UNICEF model, the basic determinants are factors within the political, economic, social, physical and environmental context at a national level. In our study, these factors were categorised as cluster- and state-level characteristics and can be summarised into two main categories: political factors and socioeconomic factors. The first category, which included only state-level characteristics, contains the central predictor: armed conflict. This variable measured intensity level/year of eruption of a conflict and was generated using the classification of conflict intensity defined by the Heidelberg Institute for International Conflict Research (HIIK) (2).

The literature also portrays the association between armed conflict and children’s nutritional status according to two different pathways. The first is direct, appearing in the form of malnourishment through a conflict’s effect on household food security and hence, children’s food consumption (3). The second pathway operates indirectly through its influence on other socio-demographic factors such as education, wealth, availability of health services and infrastructure (4,5). These two pathways were taken into consideration for selecting the other socio-demographic predictors. Accordingly, the second category of the basic determinants comprised measures at cluster- and state-level. These were variables assessing socioeconomic and residential status. The state-level variables measured state-level principle component score (PCS)^[[1]](#footnote-1)^, whereas cluster-level variables measured area of residence (urban-rural); immunisation coverage; source of water and type of sanitation (at the cluster-level); as well as cluster-level PCS^[[2]](#footnote-2)^.

The underlying determinants were combination of child and household characteristics. These were stratified into two main categories: biological factors at child-level, as well as socioeconomic and biological indicators at household-level. The former were measured by child age and gender. These factors are not specified by the UNICEF model; however, the literature emphasises their role as biological factors that may affect children’s nutritional outcomes (6). In terms of the underlying determinants at household-level, these were biological, socioeconomic and environmental factors that were expected to influence children’s nutritional status. They included mother’s age at childbirth, number of children under-5 living in the same household, household food consumption profile (FCP), as well as mother’s education, father’s education, wealth index (quintile), and risk of contamination from sources of drinking water and types of sanitation facilities (RCWS) (at household-level).

Finally, the immediate determinants were attributable only to child-level characteristics. In this study, infectious diseases, which are specified by the UNICEF model as one of the direct determinants were measured by incidence of diarrhoea in the two weeks prior to the survey.

The construction of these predictors is described below.

**The predictors**

Child-level predictors

- Age in years (a continuous variable).
- Gender (boys, girls).
- Presence of diarrhea in the two weeks before the survey (yes, no).

Household-level predictors

- Age of mother at birth (15-19, 20-29, 30-39, 40-49 years).
- Mother’s education (none, primary, secondary+).
- Father’s education (none, primary, secondary+, father not in household).
- Household wealth index quintile (poorest, second, middle, fourth, richest).
- Food consumption profile (poor consumption, borderline consumption, acceptable consumption).
- Number of children under-5 living in the same household (a continuous variable).
- Risk of contamination from water and sanitation facilities (no risk, medium risk, high risk).

Cluster-level predictors

- Area of residence (rural, urban).
- Cluster-level risk of contamination from water and sanitation facilities (no risk, medium risk, high risk).
- Cluster-level immunisation status (fully immunised, has some immunisation, never received any immunisation).
- Cluster-level principle component score defined as cluster-level socioeconomic status index.

State-level predictors

- Conflict intensity level/year of eruption (conflict-free, low intensity/2005, high intensity/2011, high intensity/2003)
- State-level principle component score defined as state-level healthcare index.

**References:**

1. UNICEF. Strategy for improved nutrition of children and women in developing countries. A UNICEF Policy Review. The United Nations Children’s Fund. New York: N.Y; 1990.

2. Barometer C. Disputes, non-violent crises, violent crises, limited wars, wars. Heidelberg Institute for International Research (HIIK). Heidelberg; 2014.

3. Agadjanian V, Prata N. Civil war and child health: regional and ethnic dimensions of child immunization and malnutrition in Angola. Social Science & Medicine. 2003;56(12):2515–27.

4. Gates S, Hegre H, Nygård HM, Strand H. Development consequences of armed conflict. World Develoment. 2012;40(9):1713–22.

5. Dodge C.P. Health implications of war in Uganda and Sudan. Social Science & Medicine. 1990;31(6):691–8.

6. Correia LL, Campos JS, Andrade FM, Machado MM, Lindsay AC, Leite ÁJ, Rocha HA, Cunha AJ. Prevalence and determinants of child undernutrition and stunting in semiarid region of Brazil. Rev Saude Publica. 2014;48(1):19–28.

1. State-level PCS is a variable that was calculated using principle component analysis (PCA) from three variables: proportion of hospitals per 100 000 population, proportion of doctors per 100 000 population and percentage of health insurance coverage. [↑](#footnote-ref-1)
2. Cluster-level PCS is a variable that was also generated using PCA from three variables: cluster-level maternal education, cluster-level parental education and cluster-level wealth index. [↑](#footnote-ref-2)
