# Supplementary material for: Armed conflict as a determinant of children malnourishment: a cross-sectional study in The Sudan
Source: BMC Public Health. 2020 Apr 19;20:532. doi: 10.1186/s12889-020-08665-x (PMC7168991; doi:10.1186/s12889-020-08665-x)
Supplement: Supplementary file 2 — Additional File 2:. Appendix. Summary statistics of the socio-demographic predictors. [file 12889_2020_8665_MOESM2_ESM.docx]

**Additional File 2**

**Appendix**

Summary statistics of the socio-demographic predictors.

**Table A1** Characteristics of children under-5

| **Characteristics** | **Overall**  *% (SE)* |
| --- | --- |
| **Child age *(year)***  Mean ± SD | 1.99±1.41 |
| **N** | **14081** |
| **Child gender** |  |
| Boy | 51.06 (0.421) |
| Girl | 48.94 (0.421) |
| **N** | **14081** |
| **Child had diarrhoea in the last two weeks prior to the survey** |  |
| Yes | 27.11 (0.376) |
| No | 72.89 (0.376) |
| **N** | **13964** |

**Table A1** Distribution of children under-5 for household-level characteristics (and number of children under-5 live in the same household)

| **Characteristics** | **Overall**  *% (SE)* |
| --- | --- |
| **Age of mother at birth *(years)*** |  |
| 15-19 | 10.15 (0.258) |
| 20-29 | 51.94 (0.427) |
| 30-39 | 32.74 (0.401) |
| 40-49 | 5.172 (0.189) |
| **N** | **13670** |
| **Mother education** |  |
| None | 46.83 (0.421) |
| Primary | 33.17 (0.397) |
| Secondary + | 19.99 (0.337) |
| **N** | **14065** |
| **Father education** |  |
| None | 36.42 (0.407) |
| Primary | 28.70 (0.383) |
| Secondary + | 22.12 (0.351) |
| Father not in the household | 12.76 (0.283) |
| **N** | **13953** |

**Table A1** *continued*

| **Characteristics** | **Overall**  *% (SE)* |
| --- | --- |
| **Household wealth index *(quintile)*** |  |
| Poorest | 23.07 (0.355) |
| Second | 26.52 (0.372) |
| Middle | 21.93 (0.349) |
| Fourth | 15.71 (0.307) |
| Richest | 12.78 (0.281) |
| **N** | **14081** |
| **Food consumption score** |  |
| Poor consumption | 4.276 (0.170) |
| Borderline consumption | 16.07 (0.310) |
| Acceptable consumption | 79.65 (0.339) |
| **N** | **14080** |
| **Number of children under-5 live in the same household** |  |
| Mean ± SD | 1.90 ± 0.78 |
| **N** | **14081** |
| **Risk of contamination from water sources and sanitation facilities** |  |
| No risk | 30.30 (0.393) |
| Medium risk | 49.30 (0.428) |
| High risk | 20.40 (0.345) |
| **N** | **13660** |

**Table A1** Distribution of children under-5 for cluster-level characteristics (and cluster-level principle component score)

| **Characteristics** | **Overall**  *% (SE)* |
| --- | --- |
| **Place of residence** |  |
| Urban | 27.06 (0.374) |
| Rural | 72.94 (0.374) |
| **N** | **14081** |
| **Cluster-level maternal education** |  |
| None | 64.08 (0.404) |
| Primary | 22.66 (0.353) |
| Secondary + | 13.26 (0.286) |
| **N** | **14081** |
| **Cluster-level paternal education** |  |
| None | 50.82 (0.421) |
| Primary | 21.48 (0.346) |
| Secondary + | 20.66 (0.341) |
| Father not in the Household | 7.038 (0.216) |
| **N** | **14081** |

**Table A1** *continued*

| **Characteristics** | **Overall**  *% (SE)* |
| --- | --- |
| **Cluster-level wealth index *(quintile)*** |  |
| Poorest | 26.57 (0.372) |
| Second | 24.05 (0.360) |
| Middle | 21.56 (0.347) |
| Fourth | 15.60 (0.306) |
| Richest | 12.22 (0.276) |
| **N** | **14081** |
| **Cluster-level risk of contamination from water sources and sanitation facilities** |  |
| No risk | 25.69 (0.368) |
| Medium risk | 54.67 (0.420) |
| High risk | 19.64 (0.335) |
| **N** | **14081** |
| **Cluster-level immunisation** |  |
| Fully immunised | 64.37 (0.409) |
| Has some immunisation | 21.70 (0.352) |
| Never received any immunisation | 13.93 (0.296) |
| **N** | **13698** |
| **Cluster-level principle component score** |  |
| Mean ± SD | -0.17 *±* 1.40 |
| **N** | **14081** |

**Table A1** State-level characteristics (children under-5)

| **Characteristics** | **Overall** |
| --- | --- |
| **Hospital per 100 000 population *(number)*** |  |
| Mean ± SD | 1.28 *±* 0.73 |
| **N** | **14081** |
| **Doctor per 100 000 population *(number)*** |  |
| Mean ± SD | 10.93 *±* 8.50 |
| **N** | **14081** |
| **Health Insured Coverage *(%)*** |  |
| Mean ± SD | 31.38 *±* 10.99 |
| **N** | **14081** |
| **State-level principle component score** |  |
| Mean ± SD | -0.14 *±* 1.34 |
| **N** | **14081** |

Mean and standard deviation are provided for continuous variables, while percentage and standard error are presented for categorical variables.
