# Supplementary material for: Armed conflict as a determinant of children malnourishment: a cross-sectional study in The Sudan
Source: BMC Public Health. 2020 Apr 19;20:532. doi: 10.1186/s12889-020-08665-x (PMC7168991; doi:10.1186/s12889-020-08665-x)
Supplement: Supplementary file 3 — Additional File 3:. Appendix. Missing data for some of the predictors and the association between missing values in outcome variables and all predictors. [file 12889_2020_8665_MOESM3_ESM.docx]

**Additional File 3**

**Appendix**

**Table A2** Percentages of missing values in the socio-demographic predictors

| **Variable Name** | n=14081  % |
| --- | --- |
| The presence of diarrhoea in the last two weeks prior to the survey | 0.83 |
| Age of mother at birth | 2.90 |
| Mother education | 0.11 |
| Father education | 0.91 |
| Food consumption profile (FCP) | 0.01 |
| Risk of contamination from source of drinking water and sanitation facility (RCWS) | 2.90 |

**Table A3** Odds ratios of missingness in underweight prevalence by conflict intensity, child-, household-, cluster- and, state-level factors

| **Variable Name** | **Model (1)**  OR (95%CI) | **Model (2)**  OR (95%CI) |
| --- | --- | --- |
| **Conflict intensity level/year of eruption**  Conflict-free (ref) |  |  |
| Low intensity/2005  **(LI/2005)** | 1.05  (0.91,1.20) | 0.67^***^  (0.57,0.78) |
| High intensity/2011  **(HI/2011)** | 0.62^***^  (0.53,0.72) | 0.49^***^  (0.41,0.59) |
| High intensity/2003  **(HI/2003)** | 1.72^***^  (1.56,1.89) | 0.99  (0.86,1.13) |
| **Child age** |  | 1.26^***^  (1.11,1.42) |
| **Child age** (squared) |  | 0.99  (0.96,1.02) |
| **Child gender**  Female (ref) |  |  |
| Male |  | 0.96  (0.87,1.05) |
| **Presence of diarrhoea**  No (ref) |  |  |
| Yes |  | 0.67^***^  (0.60,0.75) |

**Table A3** *continued*

| **Variable Name** | **Model (1)**  OR (95%CI) | **Model (2)**  OR (95%CI) |
| --- | --- | --- |
| **Age of mother at birth**  15-19 |  | 1.04  (0.89,1.22) |
| 20-29 (ref) |  |  |
| 30-39 |  | 0.94  (0.85,1.05) |
| 40-49 |  | 0.85  (0.68,1.07) |
| **Mother education**  None |  | 1.10  (0.92,1.32) |
| Primary |  | 0.95  (0.81,1.13) |
| Secondary + (ref) |  |  |
| **Father education**  None |  | 1.02  (0.87,1.21) |
| Primary |  | 1.02  (0.87,1.19) |
| Secondary + (ref) |  |  |
| Father not in household |  | 1.28^**^  (1.07,1.54) |

**Table A3** *continued*

| **Variable Name** | **Model (1)**  OR (95%CI) | **Model (2)**  OR (95%CI) |
| --- | --- | --- |
| **Household wealth index quintile**  Poorest |  | 1.64^**^  (1.19,2.25) |
| Second |  | 1.69^***^  (1.26,2.27) |
| Middle |  | 1.13  (0.87,1.47) |
| Fourth |  | 0.93  (0.73,1.18) |
| Richest (ref) |  |  |
| **Food consumption profile (FCP)**  Poor consumption |  | 1.78^***^  (1.45,2.18) |
| Borderline consumption |  | 1.32^***^  (1.17,1.49) |
| Acceptable consumption (ref) |  |  |
| **Number of children under-5 in the same household** |  | 1.00  (0.94,1.06) |

**Table A3** *continued*

| **Variable Name** | **Model (1)**  OR (95%CI) | **Model (2)**  OR (95%CI) |
| --- | --- | --- |
| **Risk of contamination from water and sanitation**  No risk (ref) |  |  |
| Medium risk |  | 1.16^+^  (0.98,1.37) |
| High risk |  | 1.00  (0.79,1.25) |
| **Area of residence**  Rural |  | 0.65^***^  (0.57,0.75) |
| Urban (ref) |  |  |
| **Principle component score (PCS) (cluster-level)** |  | 0.87^***^  (0.81,0.93) |
| **Principle component score (PCS) (state-level)** |  | 1.08^**^  (1.02,1.14) |
| **Immunisation (cluster-level)**  Fully immunised (ref) |  |  |
| Has some immunisation |  | 2.00^***^  (1.78,2.25) |
| Never received any immunisation |  | 1.49^***^  (1.30,1.71) |

**Table A3** *continued*

| **Variable Name** | **Model (1)**  OR (95%CI) | **Model (2)**  OR (95%CI) |
| --- | --- | --- |
| **Risk of contamination from water and sanitation (cluster-level)**  No risk (ref) |  |  |
| Low risk |  | 1.57^***^  (1.29,1.90) |
| High risk |  | 1.46^**^  (1.13,1.89) |
| **Intercept** | 0.21^***^  (0.19,0.22) | 0.09^***^  (0.06,0.12) |
| **Observations** | 14081 | 12685 |
| **LR chi2** | 235.788 | 898.599 |

Exponentiated coefficients; 95% confidence intervals in brackets

The dependent variable: missing in underweight prevalence vs. not missing

^+^ *p* < 0.10, ^*^ *p* < 0.05, ^**^ *p* < 0.01, ^***^ *p* < 0.001

**Model (1):** adjusted for conflict intensity.

**Model (2):** adjusted for conflict intensity + child-level variables + household-level variables + cluster-level variables + state-level variables.
