# Supplementary material for: Armed conflict as a determinant of children malnourishment: a cross-sectional study in The Sudan
Source: BMC Public Health. 2020 Apr 19;20:532. doi: 10.1186/s12889-020-08665-x (PMC7168991; doi:10.1186/s12889-020-08665-x)
Supplement: Supplementary file 4 — Additional File 4:. Appendix. The associations by individual predictors for severe and moderate underweight. [file 12889_2020_8665_MOESM4_ESM.docx]

**Additional File 4**

**Appendix**

**Table A4** Association of severe underweight with conflict status, child-, household- and cluster- and state-level characteristics

|  | **Model (1)**  OR (95%CI) | **Model (2)**  OR (95%CI) | **Model (3)**  OR (95%CI) | **Model (4)**  OR (95%CI) | **Model (5)**  OR (95%CI) |
| --- | --- | --- | --- | --- | --- |
| **Conflict intensity level/year of eruption** |  |  |  |  |  |
| Conflict-free (ref) |  |  |  |  |  |
| Low intensity/2005  **(LI/2005)** |  | 2.60^***^  (1.51-4.47) | 2.73^***^  (1.52-4.92) | 1.48^+^  (0.97-2.25) | 1.60^*^  (1.03-2.49) |
| High intensity/2011  **(HI/2011)** |  | 1.45  (0.80-2.64) | 1.53  (0.80-2.93) | 0.79  (0.51-1.25) | 0.82  (0.50-1.32) |
| High intensity/2003  **(HI/2003)** |  | 1.88^**^  (1.20-2.95) | 1.97^**^  (1.21-3.21) | 0.88  (0.61-1.27) | 0.91  (0.58-1.44) |
| **Child age** |  |  | 3.53^***^  (2.83-4.40) | 3.51^***^  (2.82-4.37) | 3.53^***^  (2.83-4.39) |

**Table A4** *continued*

|  | **Model (1)**  OR (95%CI) | **Model (2)**  OR (95%CI) | **Model (3)**  OR (95%CI) | **Model (4)**  OR (95%CI) | **Model (5)**  OR (95%CI) |
| --- | --- | --- | --- | --- | --- |
| **Child age** (squared) |  |  | 0.77^***^  (0.73-0.81) | 0.77^***^  (0.73-0.81) | 0.77^***^  (0.73-0.81) |
| **Child gender** |  |  |  |  |  |
| Female (ref) |  |  |  |  |  |
| Male |  |  | 1.16^+^  (0.99-1.37) | 1.16^+^  (0.99-1.37) | 1.17^+^  (0.99-1.37) |
| **Presence of diarrhoea** |  |  |  |  |  |
| No (ref) |  |  |  |  |  |
| Yes |  |  | 1.70^***^  (1.41-2.04) | 1.65^***^  (1.37-1.98) | 1.66^***^  (1.38-2.00) |

**Table A4** *continued*

|  | **Model (1)**  OR (95%CI) | **Model (2)**  OR (95%CI) | **Model (3)**  OR (95%CI) | **Model (4)**  OR (95%CI) | **Model (5)**  OR (95%CI) |
| --- | --- | --- | --- | --- | --- |
| **Age of mother at birth** |  |  |  |  |  |
| 15-19 |  |  |  | 0.93  (0.71-1.24) | 0.94  (0.71-1.24) |
| 20-29 (ref) |  |  |  |  |  |
| 30-39 |  |  |  | 1.01  (0.84-1.23) | 1.01  (0.84-1.22) |
| 40-49 |  |  |  | 0.98  (0.68-1.43) | 0.97  (0.67-1.42) |
| **Mother education** |  |  |  |  |  |
| None |  |  |  | 3.03^***^  (2.15-4.26) | 2.98^***^  (2.11-4.21) |
| Primary |  |  |  | 2.06^***^  (1.52-2.80) | 2.04^***^  (1.50-2.78) |
| Secondary + (ref) |  |  |  |  |  |

**Table A4** *continued*

|  | **Model (1)**  OR (95%CI) | **Model (2)**  OR (95%CI) | **Model (3)**  OR (95%CI) | **Model (4)**  OR (95%CI) | **Model (5)**  OR (95%CI) |
| --- | --- | --- | --- | --- | --- |
| **Father education** |  |  |  |  |  |
| None |  |  |  | 1.15  (0.86-1.54) | 1.13  (0.84-1.51) |
| Primary |  |  |  | 1.17  (0.89-1.53) | 1.15  (0.87-1.50) |
| Secondary + (ref) |  |  |  |  |  |
| Father not in household |  |  |  | 1.25  (0.89-1.74) | 1.23  (0.88-1.72) |

**Table A4** *continued*

|  | **Model (1)**  OR (95%CI) | **Model (2)**  OR (95%CI) | **Model (3)**  OR (95%CI) | **Model (4)**  OR (95%CI) | **Model (5)**  OR (95%CI) |
| --- | --- | --- | --- | --- | --- |
| **Household wealth index quintile** |  |  |  |  |  |
| Poorest |  |  |  | 4.15^***^  (2.43-7.07) | 3.10^***^  (1.69-5.69) |
| Second |  |  |  | 4.24^***^  (2.58-6.96) | 3.35^***^  (1.93-5.83) |
| Middle |  |  |  | 3.13^***^  (1.99-4.94) | 2.71^***^  (1.67-4.42) |
| Fourth |  |  |  | 2.21^***^  (1.46-3.36) | 2.07^***^  (1.35-3.16) |
| Richest (ref) |  |  |  |  |  |

**Table A4** *continued*

|  | **Model (1)**  OR (95%CI) | **Model (2)**  OR (95%CI) | **Model (3)**  OR (95%CI) | **Model (4)**  OR (95%CI) | **Model (5)**  OR (95%CI) |
| --- | --- | --- | --- | --- | --- |
| **Food consumption profile (FCP)** |  |  |  |  |  |
| Poor consumption |  |  |  | 0.81  (0.50-1.29) | 0.81  (0.51-1.29) |
| Borderline consumption |  |  |  | 1.10  (0.86-1.41) | 1.10  (0.86-1.41) |
| Acceptable consumption (ref) |  |  |  |  |  |
| **Number of children under-5** |  |  |  | 1.03  (0.92-1.16) | 1.03  (0.92-1.16) |

**Table A4** *continued*

|  | **Model (1)**  OR (95%CI) | **Model (2)**  OR (95%CI) | **Model (3)**  OR (95%CI) | **Model (4)**  OR (95%CI) | **Model (5)**  OR (95%CI) |
| --- | --- | --- | --- | --- | --- |
| **Risk of contamination from water and sanitation** |  |  |  |  |  |
| No risk (ref) |  |  |  |  |  |
| Medium risk |  |  |  | 1.16  (0.89-1.51) | 1.22  (0.92-1.64) |
| High risk |  |  |  | 1.34^+^  (0.95-1.89) | 1.24  (0.81-1.90) |
| **Area of residence** |  |  |  |  |  |
| Rural |  |  |  |  | 1.65^**^  (1.22-2.24) |
| Urban (ref) |  |  |  |  |  |

**Table A4** *continued*

|  | **Model (1)**  OR (95%CI) | **Model (2)**  OR (95%CI) | **Model (3)**  OR (95%CI) | **Model (4)**  OR (95%CI) | **Model (5)**  OR (95%CI) |
| --- | --- | --- | --- | --- | --- |
| **Risk of contamination from water and sanitation (cluster-level)** |  |  |  |  |  |
| No risk (ref) |  |  |  |  |  |
| Low risk |  |  |  |  | 0.77  (0.53-1.11) |
| High risk |  |  |  |  | 0.86  (0.52-1.44) |
| **Immunisation (cluster-level)** |  |  |  |  |  |
| Fully immunised (ref) |  |  |  |  |  |
| Has some immunisation |  |  |  |  | 1.25  (0.94-1.66) |
| Never received any immunisation |  |  |  |  | 0.82  (0.58-1.16) |

**Table A4** *continued*

|  | **Model (1)**  OR (95%CI) | **Model (2)**  OR (95%CI) | **Model (3)**  OR (95%CI) | **Model (4)**  OR (95%CI) | **Model (5)**  OR (95%CI) |
| --- | --- | --- | --- | --- | --- |
| **Principle component score (PCS) (cluster-level)** |  |  |  |  | 0.96  (0.84-1.10) |
| **Principle component score (PCS) (state-level)** |  |  |  |  | 0.97  (0.84-1.13) |
| **Intercept** | 0.09^***^  (0.07-0.13) | 0.06^***^  (0.05-0.09) | 0.02^***^  (0.01-0.03) | 0.003^***^  (0.001-0.006) | 0.003^***^  (0.001-0.006) |
| **State-level variance** | 0.24^*^  (0.10-0.57) | 0.10  (0.03-0.30) | 0.12  (0.04-0.35) | 0.04  (0.01-0.19) | 0.04  (0.01-0.20) |
| **Cluster-level variance** | 0.86^***^  (0.63-1.18) | 0.86^***^  (0.63-1.18) | 1.00^***^  (0.73-1.36) | 0.61^***^  (0.42-0.90) | 0.57^***^  (0.38-0.84) |
| **Household-level variance** | 1.86^***^  (1.28-2.70) | 1.86^***^  (1.28-2.70) | 2.13^***^  (1.48-3.06) | 1.95^***^  (1.34-2.85) | 1.92^***^  (1.31-2.81) |
| **State-level ICC** | 0.04 | 0.02 | 0.02 | 0.01 | 0.01 |
| **Cluster-level ICC** | 0.18 | 0.16 | 0.17 | 0.11 | 0.10 |

**Table A4** *continued*

|  | **Model (1)**  OR (95%CI) | **Model (2)**  OR (95%CI) | **Model (3)**  OR (95%CI) | **Model (4)**  OR (95%CI) | **Model (5)**  OR (95%CI) |
| --- | --- | --- | --- | --- | --- |
| **Household-level ICC** | 0.47 | 0.46 | 0.50 | 0.44 | 0.43 |
| **Observations** | 8191 | 8191 | 8191 | 8191 | 8191 |
| **LR chi2** |  | 14.657 | 169.015 | 273.180 | 281.160 |

Values are relative odds ratios; 95% confidence intervals in brackets unless stated otherwise

The dependent variable: severe underweight vs. normal

^+^ *p* < 0.10, ^*^ *p* < 0.05, ^**^ *p* < 0.01, ^***^ *p* < 0.001

**Model (1):** the empty model.

**Model (2):** adjusted for conflict intensity.

**Model (3):** adjusted for conflict intensity + child-level predictors.

**Model (4):** adjusted for conflict intensity + child-level predictors + household-level predictors.

**Model (5):** adjusted for conflict intensity + child-level predictors + household-level predictors + cluster-level predictors + state-level predictors.

**Table A5** Association of moderate underweight with conflict status, child-, household- and cluster- and state-level characteristics

|  | **Model (1)**  OR (95%CI) | **Model (2)**  OR (95%CI) | **Model (3)**  OR (95%CI) | **Model (4)**  OR (95%CI) | **Model (5)**  OR (95%CI) |
| --- | --- | --- | --- | --- | --- |
| **Conflict intensity level/year of eruption** |  |  |  |  |  |
| Conflict-free (ref) |  |  |  |  |  |
| Low intensity/2005  **(LI/2005)** |  | 1.34^*^  (1.03-1.74) | 1.33^+^  (1.00-1.78) | 0.99  (0.78-1.25) | 1.05  (0.84-1.32) |
| High intensity/2011  **(HI/2011)** |  | 1.22  (0.94-1.59) | 1.24  (0.92-1.68) | 0.90  (0.71-1.14) | 0.96  (0.76-1.20) |
| High intensity/2003  **(HI/2003)** |  | 1.27^*^  (1.03-1.56) | 1.29^*^  (1.02-1.63) | 0.83^+^  (0.68-1.02) | 0.96  (0.77-1.22) |
| **Child age** |  |  | 2.24^***^  (1.93-2.59) | 2.26^***^  (1.95-2.62) | 2.26^***^  (1.95-2.62) |
| **Child age** (squared) |  |  | 0.87^***^  (0.84-0.90) | 0.87^***^  (0.84-0.90) | 0.87^***^  (0.84-0.90) |

**Table A5** *continued*

|  | **Model (1)**  OR (95%CI) | **Model (2)**  OR (95%CI) | **Model (3)**  OR (95%CI) | **Model (4)**  OR (95%CI) | **Model (5)**  OR (95%CI) |
| --- | --- | --- | --- | --- | --- |
| **Child gender** |  |  |  |  |  |
| Female (ref) |  |  |  |  |  |
| Male |  |  | 1.11^+^  (0.99-1.24) | 1.10^+^  (0.99-1.23) | 1.11^+^  (0.99-1.24) |
| **Presence of diarrhoea** |  |  |  |  |  |
| No (ref) |  |  |  |  |  |
| Yes |  |  | 1.42^***^  (1.25-1.62) | 1.41^***^  (1.24-1.60) | 1.41^***^  (1.24-1.60) |

**Table A5** *continued*

|  | **Model (1)**  OR (95%CI) | **Model (2)**  OR (95%CI) | **Model (3)**  OR (95%CI) | **Model (4)**  OR (95%CI) | **Model (5)**  OR (95%CI) |
| --- | --- | --- | --- | --- | --- |
| **Age of mother at birth** |  |  |  |  |  |
| 15-19 |  |  |  | 0.93  (0.76-1.13) | 0.92  (0.76-1.12) |
| 20-29 (ref) |  |  |  |  |  |
| 30-39 |  |  |  | 1.03  (0.90-1.17) | 1.04  (0.91-1.18) |
| 40-49 |  |  |  | 0.74^*^  (0.56-0.98) | 0.75^*^  (0.57-0.99) |
| **Mother education** |  |  |  |  |  |
| None |  |  |  | 1.22^+^  (0.99-1.51) | 1.17  (0.94-1.44) |
| Primary |  |  |  | 1.12  (0.93-1.34) | 1.10  (0.91-1.32) |
| Secondary + (ref) |  |  |  |  |  |

**Table A5** *continued*

|  | **Model (1)**  OR (95%CI) | **Model (2)**  OR (95%CI) | **Model (3)**  OR (95%CI) | **Model (4)**  OR (95%CI) | **Model (5)**  OR (95%CI) |
| --- | --- | --- | --- | --- | --- |
| **Father education** |  |  |  |  |  |
| None |  |  |  | 1.18^+^  (0.98-1.44) | 1.13  (0.93-1.37) |
| Primary |  |  |  | 1.12  (0.94-1.33) | 1.10  (0.92-1.30) |
| Secondary + (ref) |  |  |  |  |  |
| Father not in household |  |  |  | 0.91  (0.73-1.14) | 0.89  (0.71-1.11) |

**Table A5** *continued*

|  | **Model (1)**  OR (95%CI) | **Model (2)**  OR (95%CI) | **Model (3)**  OR (95%CI) | **Model (4)**  OR (95%CI) | **Model (5)**  OR (95%CI) |
| --- | --- | --- | --- | --- | --- |
| **Household wealth index quintile** |  |  |  |  |  |
| Poorest |  |  |  | 2.76^***^  (1.99-3.84) | 1.97^***^  (1.35-2.87) |
| Second |  |  |  | 2.43^***^  (1.80-3.29) | 1.83^***^  (1.31-2.57) |
| Middle |  |  |  | 2.05^***^  (1.56-2.69) | 1.70^***^  (1.27-2.28) |
| Fourth |  |  |  | 1.73^***^  (1.36-2.20) | 1.58^***^  (1.24-2.01) |
| Richest (ref) |  |  |  |  |  |

**Table A5** *continued*

|  | **Model (1)**  OR (95%CI) | **Model (2)**  OR (95%CI) | **Model (3)**  OR (95%CI) | **Model (4)**  OR (95%CI) | **Model (5)**  OR (95%CI) |
| --- | --- | --- | --- | --- | --- |
| **Food consumption profile (FCP)** |  |  |  |  |  |
| Poor consumption |  |  |  | 1.02  (0.74-1.41) | 1.05  (0.76-1.45) |
| Borderline consumption |  |  |  | 0.96  (0.81-1.15) | 0.98  (0.82-1.16) |
| Acceptable consumption (ref) |  |  |  |  |  |
| **Number of children under-5** |  |  |  | 0.98  (0.91-1.06) | 0.98  (0.91-1.07) |
| **Risk of contamination from water and sanitation** |  |  |  |  |  |
| No risk (ref) |  |  |  |  |  |
| Medium risk |  |  |  | 1.02  (0.86-1.21) | 1.02  (0.85-1.24) |
| High risk |  |  |  | 1.16  (0.92-1.46) | 1.08  (0.81-1.45) |

**Table A5** *continued*

|  | **Model (1)**  OR (95%CI) | **Model (2)**  OR (95%CI) | **Model (3)**  OR (95%CI) | **Model (4)**  OR (95%CI) | **Model (5)**  OR (95%CI) |
| --- | --- | --- | --- | --- | --- |
| **Area of residence** |  |  |  |  |  |
| Rural |  |  |  |  | 1.56^***^  (1.29-1.88) |
| Urban (ref) |  |  |  |  |  |
| **Risk of contamination from water and sanitation (cluster-level)** |  |  |  |  |  |
| No risk (ref) |  |  |  |  |  |
| Low risk |  |  |  |  | 0.90  (0.71-1.13) |
| High risk |  |  |  |  | 0.90  (0.64-1.27) |

**Table A5** *continued*

|  | **Model (1)**  OR (95%CI) | **Model (2)**  OR (95%CI) | **Model (3)**  OR (95%CI) | **Model (4)**  OR (95%CI) | **Model (5)**  OR (95%CI) |
| --- | --- | --- | --- | --- | --- |
| **Immunisation (cluster-level)** |  |  |  |  |  |
| Fully immunised (ref) |  |  |  |  |  |
| Has some immunisation |  |  |  |  | 1.08  (0.89-1.30) |
| Never received any immunisation |  |  |  |  | 0.99  (0.80-1.24) |
| **Principle component score (PCS) (cluster-level)** |  |  |  |  | 0.92^*^  (0.84-1.00) |
| **Principle component score (PCS) (state-level)** |  |  |  |  | 1.05  (0.98-1.13) |
| **Intercept** | 0.27^***^  (0.24-0.30) | 0.23^***^  (0.20-0.27) | 0.09^***^  (0.07-0.11) | 0.05^***^  (0.03-0.06) | 0.04^***^  (0.03-0.06) |
| **State-level variance** | 0.03  (0.01-0.10) | 0.01  (0.001-0.10) | 0.02  (0.003-0.01) | 0.003  (5e^-06^-1.53) | 7.66e^-35^  - |

**Table A5** *continued*

|  | **Model (1)**  OR (95%CI) | **Model (2)**  OR (95%CI) | **Model (3)**  OR (95%CI) | **Model (4)**  OR (95%CI) | **Model (5)**  OR (95%CI) |
| --- | --- | --- | --- | --- | --- |
| **Cluster-level variance** | 0.31^***^  (0.23-0.43) | 0.31^***^  (0.23-0.43) | 0.36^***^  (0.27-0.50) | 0.25^***^  (0.17-0.36) | 0.22^***^  (0.14-0.33) |
| **Household-level variance** | 0.48^***^  (0.26-0.86) | 0.47^***^  (0.26-0.86) | 0.59^***^  (0.35-1.01) | 0.59^***^  (0.35-1.01) | 0.57^***^  (0.33-0.98) |
| **State-level ICC** | 0.01 | 0.003 | 0.004 | 0.001 | 1.88e-35 |
| **Cluster-level ICC** | 0.08 | 0.08 | 0.09 | 0.06 | 0.05 |
| **Household-level ICC** | 0.20 | 0.19 | 0.23 | 0.20 | 0.20 |
| **Observations** | 9080 | 9080 | 9080 | 9080 | 9080 |
| **LR chi2** |  | 7.702 | 216.864 | 303.887 | 329.704 |

Values are relative odds ratios; 95% confidence intervals in brackets unless stated otherwise

The dependent variable: moderate underweight vs. normal

^+^ *p* < 0.10, ^*^ *p* < 0.05, ^**^ *p* < 0.01, ^***^ *p* < 0.001

**Model (1):** the empty model.

**Model (2):** adjusted for conflict intensity.

**Model (3):** adjusted for conflict intensity + child-level predictors.

**Model (4):** adjusted for conflict intensity + child-level predictors + household-level predictors.

**Model (5):** adjusted for conflict intensity + child-level predictors + household-level predictors + cluster-level predictors + state-level predictors.
